# Discrepancies among Scopus, Web of Science, and PubMed coverage of funding information in medical journal articles

**DOI:** 10.5195/jmla.2018.181

**Published:** 2018-01-02

**Authors:** Peter Kokol, Helena Blažun Vošner

## Abstract

**Objective:**

The overall aim of the present study was to compare the coverage of existing research funding information for articles indexed in Scopus, Web of Science, and PubMed databases.

**Methods:**

The numbers of articles with funding information published in 2015 were identified in the three selected databases and compared using bibliometric analysis of a sample of twenty-eight prestigious medical journals.

**Results:**

Frequency analysis of the number of articles with funding information showed statistically significant differences between Scopus, Web of Science, and PubMed databases. The largest proportion of articles with funding information was found in Web of Science (29.0%), followed by PubMed (14.6%) and Scopus (7.7%).

**Conclusion:**

The results show that coverage of funding information differs significantly among Scopus, Web of Science, and PubMed databases in a sample of the same medical journals. Moreover, we found that, currently, funding data in PubMed is more difficult to obtain and analyze compared with that in the other two databases.

## INTRODUCTION

As funding is a significant factor enabling research projects [1], research institutions compete for grants on a routine basis [2]. Institutions with more grant funding have a greater ability to hire eminent researchers, provide access to advanced technology and research equipment, cooperate in major international scientific networks, gather new knowledge at top conferences, and/or hire leading external organizations to support the preparation of competitive project proposals. Subsequently, such institutions perform better research, publish more high-quality publications, and attract more citations [3, 4].

In previous studies, Boyack showed that articles resulting from large grants were cited more than those from small grants [5], and Wang and Shapira found that funded publications had more impact in terms of journal rankings and numbers of citations [6]. Consequently, knowledge about funding patterns found in funding statements could be of vital importance to researchers who are seeking grants and others who are interested in assessing the impact and outcomes of funding [7]. Funding patterns can be used for strategic intelligence applications, such as mapping funding landscapes and generating funding organization portfolios [8], and can be used to identify top-funded topics and themes, acquire lists of funding organizations, and locate successful grant holders for possible collaboration.

Some research funding information can be obtained directly from funding agency reports or databases. For example, in their analysis of the impact of federal life sciences funding for university research and development programs, Blume-Kohout, Kumar, and Sood used datasets from the US National Science Foundation and US National Institutes of Health to measure funding expenditures [9]. However, not all such data are easily accessible, if at all. Hence, Wang and Shapira proposed the possibility of analyzing funding information acknowledgments found in bibliographical databases [10]. However, Rigby warned that the uncritical use of funding information found in bibliographic databases might lead to bias in interpreting search results [11]. For instance, Tang et al. noted limitations in the funding information found in Web of Science (WoS) (Thomson Reuters, USA), with English language articles showing greater coverage than articles in other languages and engineering, as well as biomedical articles showing greater coverage than social sciences and humanities articles [12].

Of the more than 100 bibliometric databases, only WoS, Scopus (Elsevier, Netherlands), and PubMed (National Library of Medicine, United States) databases provide funding information for indexed articles. Whereas Scopus and WoS are general subscription databases, PubMed is a publicly accessible database covering mostly biomedical literature. Of these, Scopus indexes the largest number of publications. The overall aim of this study was to determine differences between WoS, Scopus, and PubMed databases in terms of the accessibility, scope, and volume of funding information for indexed articles.

## METHODS

The authors analyzed funding information for articles published in three prestigious families of journals indexed in WoS, Scopus, and PubMed databases: *The Lancet, Journal of American Medical Association (JAMA),* and *British Medical Journal (BMJ).*

Funding information can be obtained from various fields in the three databases. Funding information can appear in the funding organization, grant number, and funding acknowledgment text fields in WoS; the funding sponsor and grant acronym fields of Scopus; and the grant number and publication type fields of PubMed. Preliminary analysis showed that the field identifying the largest number of funded articles (FAs) in a database also covers all FAs identified by the other fields in that database. Hence, we selected the funding organization field for WoS, the funding sponsor field for Scopus, and the grant number field for PubMed. To form a list of all possible funding organizations and sponsors, we used a wildcard character (*) to represent a string of characters of any length. Two corpuses (one for FAs and one for all articles) from each database were created for articles published in 2015 (Table 1).

**Table 1:** Search strings used to retrieve articles with funding information

Database	Search string
Web of Science (WoS)	so = (jama* or BMJ* or Lancet*) and py = 2015 and FO = (a* or b* or c* or d* or e* or f* or g* or h* or i* or j* or k* or l* or m* or n* or o* or p* or q* or r* or s* or t* or u* or v* or z* or x* or y* or w* or 1* or 2* or 3* or 4* or 5* or 6* or 7* or 8* or 9* or 0*)
Scopus	SRCTITLE(Lancet or BMJ or jama) and pubyear = 2015 and fund-sponsor = (a* or b* or c* or d* or e* or f* or g* or h* or i* or j* or k* or l* or m* or n* or o* or p* or q* or r* or s* or t* or u* or v* or z* or x* or y* or w* or 1* or 2* or 3* or 4* or 5* or 6* or 7* or 8* or 9* or 0*)
PubMed	(((((((((((((((((((((((((((((((((((((((((((“BMJ open”[Journal]) OR “BMJ (Clinical research ed.)”[Journal]) OR “BMJ case reports”[Journal]) OR “BMJ quality & safety”[Journal]) OR “BMJ clinical evidence”[Journal]) OR “BMJ supportive & palliative care”[Journal]) OR “BMJ quality improvement reports”[Journal]) OR “BMJ open diabetes research & care”[Journal]) OR “BMJ open respiratory research”[Journal]) OR “BMJ open sport & exercise medicine”[Journal]) OR “BMJ open gastroenterology”[Journal]) OR “BMJ innovations”[Journal]) OR “BMJ global health”[Journal]) OR “BMJ outcomes”[Journal]) OR “Lancet (London, England)”[Journal]) OR “The Lancet. Oncology”[Journal]) OR “The Lancet. Infectious diseases”[Journal]) OR “The Lancet. Neurology”[Journal]) OR “The Lancet. Respiratory medicine”[Journal]) OR “The Lancet. Diabetes & endocrinology”[Journal]) OR “The Lancet. Global health”[Journal]) OR “The Lancet. Psychiatry”[Journal]) OR “The Lancet. HIV”[Journal]) OR “The Lancet. Hematology”[Journal]) OR “The Lancet. Gastroenterology & hepatology”[Journal]) OR “JAMA”[Journal]) OR “JAMA internal medicine”[Journal]) OR “JAMA dermatology”[Journal]) OR “JAMA ophthalmology”[Journal]) OR “JAMA neurology”[Journal]) OR “JAMA surgery”[Journal]) OR “JAMA pediatrics”[Journal]) OR “JAMA otolaryngology-- head & neck surgery”[Journal]) OR “JAMA psychiatry”[Journal]) OR “JAMA oncology”[Journal]) OR “JAMA facial plastic surgery”[Journal]) OR “JAMA cardiology”[Journal]) AND (“2015”[Date - Publication] : “2015”[Date - Publication])))))

WoS and Scopus databases allowed us to directly extract the number of all articles and number of FAs for each journal using built-in services. However, for PubMed, we first exported the corpus to BibTex and then to MS Excel (Microsoft, USA), in which we performed the analysis using the crosstab function.

We found that some *BMJ* journals were not indexed by all three databases, so these journals were omitted from subsequent analyses. The numbers of articles published in the remaining journals were compared between databases using paired Student’s *t*-tests. Finally, we performed an analysis of the document types of FAs.

## RESULTS

We identified 28 journals containing 16,927 articles in Scopus, 25 journals containing 14,494 articles in WoS, and 34 journals containing 16,967 articles in PubMed. Of these articles, there were 1,306 FAs in Scopus, 4,206 FAs in WoS, and 2,482 FAs in PubMed.

Although there were large differences among databases in both the number of all articles and the number of FAs in individual journals, the largest variations among databases were seen in the numbers of FAs. WoS identified the largest percentage of FAs for all journals. Scopus identified the lowest percentage of FAs for all journals, except *BMJ Open,* for which a lower percentage of FAs was identified in PubMed. The largest difference among databases was observed for the journal *Lancet Diabetes & Endocrinology,* for which there were 0.4% of identified FAs in Scopus, 23.6% in WoS, and 17.3% in PubMed. Overall, the percentage of all identified FAs was 29.0% in WoS, 14.6% in PubMed, and 7.7% in Scopus.

After removing the journals that were not identified in all 3 databases, paired Student’s *t*-tests showed significant differences in the numbers of FAs identified between Scopus and WoS (*t*(23)=−3.120, *p*<0.01) and between Scopus and PubMed (*t*(23)=−4.588, *p*<0.01). There was no significant difference between WoS and PubMed. Although there were also differences between the numbers of all identified articles in Scopus, WoS, and PubMed, these differences were not statistically significant (*p*>0.05).

The analysis of document types revealed that all FAs identified in Scopus were classified as articles. The most frequent types of FAs in WoS were articles (73%), editorials (12%), reviews (8%), and letters (6%), whereas the most frequent types of FAs in PubMed were articles (75%), reviews (7%), letters (6%), and editorials (3%).

## DISCUSSION

Funded research is reportedly of higher quality and cited more often [4]. Knowledge of funding patterns can enhance a researcher’s likelihood of receiving funding, and these funding patterns can be acquired through bibliometric analysis of published journal articles. Although Scopus, WoS, and PubMed provide funding information, it is unclear whether the quantity of this information for medical journal articles is equivalent, meaning that selecting the “wrong” database can lead to biased analyses and misleading results.

**Table 2:** Numbers of all articles and funded articles (FAs) published in 2015 for three families of journals

Title	Scopus			WoS			PubMed		
		
	All articles	Funded articles (FAs)	%	All articles	FAs	%	All articles	FAs	%
*Lancet*	2,056	55	2.70%	2,000	363	18.20%	1,926	281	14.60%
*Lancet Respiratory Medicine*	340	9	2.70%	301	74	24.60%	330	48	14.00%
*Lancet Psychiatry*	286	5	1.80%	369	127	34.40%	339	59	17.40%
*Lancet Oncology*	666	11	1.70%	647	177	27.40%	694	87	12.60%
*Lancet Neurology*	300	8	2.70%	216	102	47.20%	302	65	21.50%
*Lancet Infectious Diseases*	449	7	1.60%	451	141	31.30%	452	105	23.20%
*Lancet HIV*	166	6	3.60%	140	59	42.10%	145	46	31.70%
*Lancet Hematology*	120	11	9.10%	123	44	35.80%	122	21	17.20%
*Lancet Global Health*	239	7	2.90%	272	111	40.80%	235	59	25.10%
*Lancet Diabetes & Endocrinology*	283	1	0.40%	301	71	23.60%	277	48	17.30%
*JAMA Surgery*	313	31	9.90%	307	67	21.80%	304	42	13.80%
*JAMA Psychiatry*	237	95	40.10%	226	154	68.10%	224	105	46.90%
*JAMA Pediatrics*	327	61	18.70%	313	132	42.10%	306	104	33.90%
*JAMA Otolaryngology Head and Neck Surgery*	225	17	7.60%	223	52	23.30%	220	34	15.50%
*JAMA Ophthalmology*	447	69	15.40%	427	169	39.60%	424	106	25.00%
*JAMA Oncology*	248	57	22.90%	76	32	42.10%	273	77	28.20%
*JAMA Neurology*	331	114	34.40%	320	161	50.30%	318	117	36.80%
*JAMA Journal of the American Medical Association*	1,489	172	11.60%	1,584	289	18.20%	1,301	212	16.30%
*JAMA Internal Medicine*	703	103	14.70%	654	206	31.50%	652	159	24.40%
*JAMA Facial Plastic Surgery*	104	1	0.90%	101	16	15.80%	97	5	5.20%
*JAMA Dermatology*	423	36	8.50%	384	99	25.80%	348	48	13.80%
*BMJ Supportive and Palliative Care*	84	1	1.20%	94	16	17.00%	223	22	9.90%
*BMJ Quality and Safety*	130	20	15.40%	164	62	37.80%	254	49	19.30%
*BMJ Open*	1,362	316	23.20%	1,475	1,206	81.80%	1,473	339	23.00%
*BMJ Online*	2,002	73	N/A	NI*	NI*	N/A	NI*	NI*	N/A
*BMJ Clinical Research Edition*	1,608	14	N/A	NI*	NI*	N/A	NI*	NI*	N/A
*BMJ Case Reports*	1,989	6	N/A	NI*	NI*	N/A	1,994	20	N/A
*BMJ other*	NI*	NI*	N/A	NI*	NI*	N/A	406	44	N/A
*BMJ British Medical Journal*	NI*	NI*	N/A	3,326	276	N/A	3,328	180	N/A
*Total*	16,927	1,306	7.70%	14,494	4,206	29.00%	16,967	2,482	14.60%

* NI=no information.

Here, we performed a bibliometric analysis of funding information provided by Scopus, WoS, and PubMed for articles published in a sample of prestigious medical journals. Such analyses can help to identify differences between bibliographic databases, select the most appropriate database, or reveal limitations of particular databases that enable critical assessment of the quality of reported funding patterns.

We found a significant difference in the number of identified FAs between Scopus, WoS, and PubMed databases, with WoS identifying the largest number of FAs. A previous study of 7,510 publications reporting UK cancer research in 2011 showed that WoS identified approximately 93% of funding data correctly, whereas PubMed correctly identified less than 50% of funding data. This same study also revealed the existence of a small number of publications in the WoS database that claimed funding but did not actually receive it [8]. Additionally, since 2008, WoS has been collecting funding information by indexing the source text directly from the journal articles [13], which might partially explain why more FAs were identified in WoS than in the other 2 databases.

A recent study reported that articles and reviews were the most consistently covered publication types that contained funding information in WoS [14]. Articles were also the most common publication type identified in our study. However, in addition to articles, we found considerable numbers of editorials and letters that contained funding information in WoS and PubMed. Hence, the notable presence of editorials and letters among funded publication types might also partially explain differences among the three databases.

Our study shows that coverage of funding information differs significantly between the Scopus, WoS, and PubMed databases for a sample of prestigious medical journals. Consequently, the selection of a bibliographic database in an analysis of research funding might bias the results of that analysis. Moreover, funding data in the PubMed database is, from an analytical point-of-view, harder to obtain and analyze compared with that in the Scopus or WoS databases. However, access to PubMed is free, in contrast to the other two databases that require subscriptions. We would, therefore, advise administrators, librarians, and investigators searching for funding information on particular research topics or for particular institutions to use all three databases to obtain more complete information. If only one database is available, we recommend using WoS. If the lack of a subscription prevents access to WoS, PubMed is a viable alternative.

We acknowledge the limitation that our study was performed on a sample of medical journals for document types published in 2015, meaning that the selection of a different sample of medical journals or publication year could lead to different results and conclusions. Another limitation is that, due to the large sample size, we were not able to compare our results with a gold standard, such as funding agency reports. However, to the best of our knowledge, this is the first bibliometric study comparing funding information acknowledged in medical journal articles that are indexed by Scopus, WoS, and PubMed databases.
